# Fasciite nécrosante de la paroi thoracique compliquant un empyème

**DOI:** 10.11604/pamj.2013.16.108.2616

**Published:** 2013-11-20

**Authors:** Narindra Njarasoa Mihaja Razafimanjato, Lalaina Elianah Rasoamampianina, Manjakaniaina Ravoatrarilandy, Auberlin Felantsoa Rakototiana, Francis Allen Hunald, Luc Hervé Samison, Hanitrala Jean Louis Rakotovao

**Affiliations:** 1 Service de Chirurgie Thoracique CHU/JRA BP: 4150 CP: 101 Antananarivo, Madagascar; 2 Service de Réanimation Chirurgicale CHU/JRA BP: 4150 CP: 101 Antananarivo, Madagascar; 3 Service de Chirurgie Viscérale CHU/JRA BP: 4150 CP: 101 Antananarivo, Madagascar

**Keywords:** Débridement chirurgical, Fasciite nécrosante, Incisions multiples, Madagascar, Paroi thoracique, Streptocoque, Vacuum Assisted Closure^®^, surgical debridement, necrotizing fasciitis, multiple incisions, Madagascar, chest wall, streptococcus, Vacuum Assisted Closure^®^

## Abstract

La fasciite nécrosante est une infection sévère, rapidement progressive, mutilante, souvent fatale du tissu sous cutané et du fascia profond. Les auteurs rapportent et discutent un rare cas de fasciite nécrosante de la paroi thoracique antérieure secondaire à une pleurésie purulente chez un diabétique. Un diagnostic précoce avec incisions multiples chirurgicales et une antibiothérapie adaptée par voie veineuse diminuent la morbidité et la mortalité causées par cette pathologie.

## Introduction

La fasciite nécrosante est une infection due à des germes aérobie et anaérobie dits «mangeurs de chair» qui touche les tissus sous cutanés et le fascia profond [1]. C'est une pathologie mutilante rare qui peut compromettre le pronostic vital par ses complications septicémiques avec un taux de mortalité élevée situé entre 30 à 76% [2]. L'atteinte de la paroi thoracique est exceptionnelle [3]. Hippocrate décrivait pour la première fois cette affection fatale au Vème siècle avant JC et le terme fasciite nécrosante était introduit par Wilson en 1952 [4]. Les auteurs décrivent ici un cas de fasciite nécrosante de la paroi thoracique secondaire à un empyème et discutent ses aspects cliniques, thérapeutiques et évolutifs à travers une revue de la littérature.

## Patient et observation

**Figure 1:**
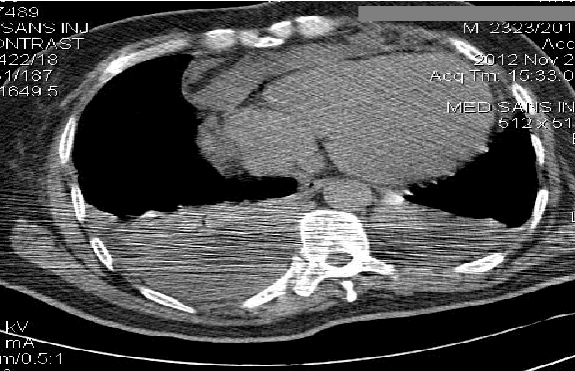
TDM thoracique sans injection de produit de contraste. Il existe un épanchement pleural bilatéral associé à une in'ltration tissulaire de la paroi

**Figure 2:**
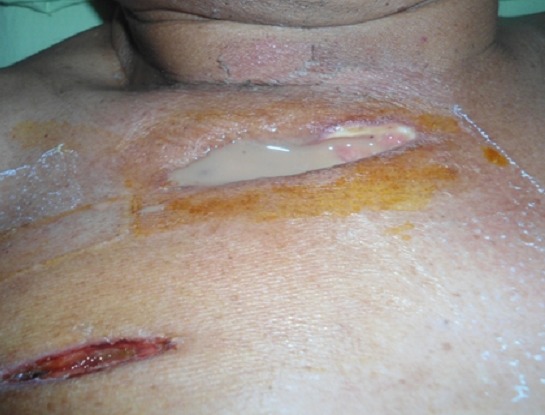
Une incision étagée et tunnelisée de la paroi thoracique antérieure

Un patient de 61 ans, diabétique de type 2 était hospitalisé en service de réanimation pour prise en charge d'un choc septique d'apparition brutale. Le bilan à l'entrée retrouvait un tableau de détresse respiratoire avec état de choc nécessitant une ventilation mécanique et un support aminérgique. Une triple antibiothérapie probabiliste associant une céphalosporine 3G, un nitro-imidazolé et aminoside était débutée. L'examen clinique retrouvait un syndrome d’épanchement pleural liquidien bilatéral et une infiltration avec collection sous cutanée de l'hémithorax homolatéral. Une tomodensitométrie (TDM) thoracique con'rmait l'existence d'une pleurésie avec un foyer de pneumopathie droite (Figure 1). Deux incisions tunnélisées étaient pratiquées avec débridement des plans sous-cutanés complétées par un drainage thoracique (Figure 2). Les prélèvements bactériologiques effectués n’étaient pas contributifs. En quelques jours, l’état général s'améliorait avec une normalisation des signes infectieux, un sevrage de la ventilation mécanique et extubation. Un pansement aspiratif était mis en place parallèlement à l'antibiothérapie. Après deux semaines de ce traitement, le patient présentait un état général satisfaisant avec une disparition complète des signes infectieux.

## Discussion

La fasciite nécrosante est une dermohypodermite bactérienne nécrosante atteignant par dé'nition le fascia super'cialis associé ou non à une atteinte musculaire (myonécrose, gangrène gazeuse) [3, 5]. Rarement rencontrée dans notre pratique, il n'existe que très peu de données concernant l’épidémiologie des fasciites nécrosantes [5]. Pour les infections invasives à streptocoque A, il est décrit de 5 à 10% des formes avec fasciites aux USA et au Canada [3, 5].

*Le Streptocoque-hémolytique* du groupe A (fasciite streptococcique) et le *Clostridium perfringens* (gangrène gazeuse) sont les pathogènes à l'origine de cette pathologie [1, 3]. Elle est le plus souvent polymicrobienne (40 à 90% des cas) chez les patients présentant des facteurs de risque [6]. L'infection débute par une nécrose de l'hypoderme avec thrombose vasculaire. La nécrose s’étend secondairement à l'aponévrose super'cielle sous-jacente puis secondairement au derme [3]. Les germes secrètent des enzymes responsables de la nécrose liquéfactive du fascia superficialis et des toxines qui se propagent dans tous l'organisme à l'origine de la septicémie [4]. Les facteurs de risque reconnus sont: l’âge > 50 ans, le diabète comme le cas de notre patient (25 à 30% des cas), les troubles vasculaires périphériques (36%), l'alcoolisme chronique et la toxicomanie (15 à 20%), l'immunodépression (cancers, traitements immunosuppresseurs, chimiothérapie) [3, 6]. Les AINS sont souvent retrouvés comme facteur aggravant. Une étude en pédiatrie a retrouvé un risque relatif à 11 de fasciite lors de l'utilisation d'ibuprofène chez l'enfant lors de varicelle [5]. Les formes thoraciques isolées sont extrêmement rare [6] dont les cas rapportés sont secondaires à un drainage thoracique, à une chirurgie pulmonaire ou ‘sophagienne ou à partir de pleurésie purulente identique à notre cas [7].

La présentation clinique des fasciites nécrosantes est très souvent parlante. La douleur initialement est au premier plan sans forcément des signes cutanés, associée à une ‘èvre élevée [5]. A un stade avancé, apparait des signes de sepsis, des lésions cutanées (érythème, lésion bulleuse hémorragique, gangrène gazeuse) avec une hypoesthésie [5, 7].

Les examens biologiques standards ont un double intérêt: évaluer le retentissement général du sepsis grave sur les différents organes et établir un score diagnostic appelé LRINEC (Laboratory Risk Indicator for Necrotizing Fasciitis) (Tableau 1). Un score plus de 8 est fortement prédictive d'une fasciite nécrosante (VPP= 93.4%) [4, 5]. La culture bactériologique des tissus infectés est utile pour adapter l'antibiothérapie [1]. Les radiographies standard sont souvent plus sensibles que l'examen clinique pour détecter du gaz dans les parties molles Les examens de référence sont le scanner spiralé et l'IRM permettant de voir un épaississement des fascias, une hétérogénéité de la graisse et la présence de gaz [5]. Ces examens permettent aussi un bilan d'extension des lésions qui est bien corrélé à la chirurgie.

**Tableau 1:** Score de LRINEC (Laboratory Risk Indicator for Necrotizing Fasciitis)

Variable	Score
**C- reactive Protein (mg/1)**	
<150	0
150 or more	4
**Total white cell count (per mm** ^**3**^ **)**	
<15	0
15-25	1
>25	2
**Hemoglobin (g/dll)**	
>13.5	0
11-13.5	1
<11	2
**Sodium (mmol/1)**	
135 or more	0
<135	2
**Creatinin (mmol/1)**	
141 or less	0
>41	2
**Glucose (mmol/1)**	
10 or less	0
>10	1

Le traitement de la fasciite nécrosante consiste à un débridement précoce et complet des tissus infectés et nécrosé afin de limiter l'extension du processus infectieux [5, 6]. La chirurgie réparatrice ne peut être envisagée qu'après cicatrisation et contrôle de l'infection [1]. Dans notre cas, deux incisions étagées et tunnélisées étaient réalisés comme le cas rapporté par HUNALD et al. [8]. Elles permettent les nettoyages quotidiens tout en évitant ainsi le vaste débridement qui imposerait une reconstruction ultérieure [3].

Aucune étude randomisée n'a prouvé l'efficacité de l'oxygénothérapie hyperbare (OHB) dans la prise en charge des fasciites nécrosantes. Seules les données expérimentales sont en faveur de l'OHB dans la gangrène gazeuse [5, 9]. L'utilisation complémentaire du VAC (Vacuum Assisted Closure) dans cette indication parait prometteuse pour accélérer la cicatrisation. Les résultats obtenus sont proches de ceux observés avec un système d'aération décrit par Kostantinov [3].

L'antibiothérapie est le deuxième pilier de la prise en charge des fasciites nécrosantes [5]. Les antibiotiques sont le plus souvent complémentaires du traitement chirurgical car la pénétration locale est insuffisante du fait des thromboses vasculaires responsables de la nécrose des plans profonds. L'objectif du traitement antibiotique est de limiter la progression de l'infection [3, 5, 10]. La prise en charge intensive de la réanimation du choc septique est bien entendu un objectif fondamental. La corticothérapie, même aux posologies de l'opothérapie substitutive ainsi que la place de la protéine C activée sont discutées compte tenu des problèmes de bourgeonnement et des problèmes hémorragiques. La nutrition entérale doit être précoce et hypercalorique (40 à 45 kcal/.kg/.j) a'n de favoriser la cicatrisation. Il peut être proposé de la glutamine et de l'immunoglobinothérapie intraveineuse par analogie à la prise en charge des grands brûlés, mais il n'existe pas d’étude dans les fasciites nécrosantes [1, 5]. Son pronostic dépend deux principaux facteurs: la précocité du diagnostic et de la prise en charge initiale. Un délai inférieur ou supérieur à 24h entre le diagnostic et la chirurgie est associé à un taux de mortalité de 36% et 70% respectivement [3].

## Conclusion

Les fasciites nécrosantes sont des infections graves, dont l'appellation est maintenant dermohypodermites bactériennes nécrosantes. Sa prise en charge doit être précoce multidisciplinaire entre chirurgien, réanimateur et infectiologue pour améliorer son pronostic dramatique.
